# Mitral valve surgery for mitral regurgitation caused by Libman-Sacks endocarditis: a report of four cases and a systematic review of the literature

**DOI:** 10.1186/1749-8090-5-13

**Published:** 2010-03-23

**Authors:** Wobbe Bouma, Theo J Klinkenberg, Iwan CC van der Horst, Inez J Wijdh-den Hamer, Michiel E Erasmus, Marc Bijl, Albert JH Suurmeijer, Felix Zijlstra, Massimo A Mariani

**Affiliations:** 1 Department of Cardiothoracic Surgery, University Medical Center Groningen, the Netherlands; 2 Department of Cardiology, University Medical Center Groningen, the Netherlands; 3 Department of Rheumatology and Clinical Immunology, University Medical Center Groningen, the Netherlands; 4 Department of Pathology, University Medical Center Groningen, the Netherlands

## Abstract

Libman-Sacks endocarditis of the mitral valve was first described by Libman and Sacks in 1924. Currently, the sterile verrucous vegetative lesions seen in Libman-Sacks endocarditis are regarded as a cardiac manifestation of both systemic lupus erythematosus (SLE) and the antiphospholipid syndrome (APS). Although typically mild and asymptomatic, complications of Libman-Sacks endocarditis may include superimposed bacterial endocarditis, thromboembolic events, and severe valvular regurgitation and/or stenosis requiring surgery. In this study we report two cases of mitral valve repair and two cases of mitral valve replacement for mitral regurgitation (MR) caused by Libman-Sacks endocarditis. In addition, we provide a systematic review of the English literature on mitral valve surgery for MR caused by Libman-Sacks endocarditis. This report shows that mitral valve repair is feasible and effective in young patients with relatively stable SLE and/or APS and only localized mitral valve abnormalities caused by Libman-Sacks endocarditis. Both clinical and echocardiographic follow-up after repair show excellent mid- and long-term results.

## Introduction

**Figure 1:**
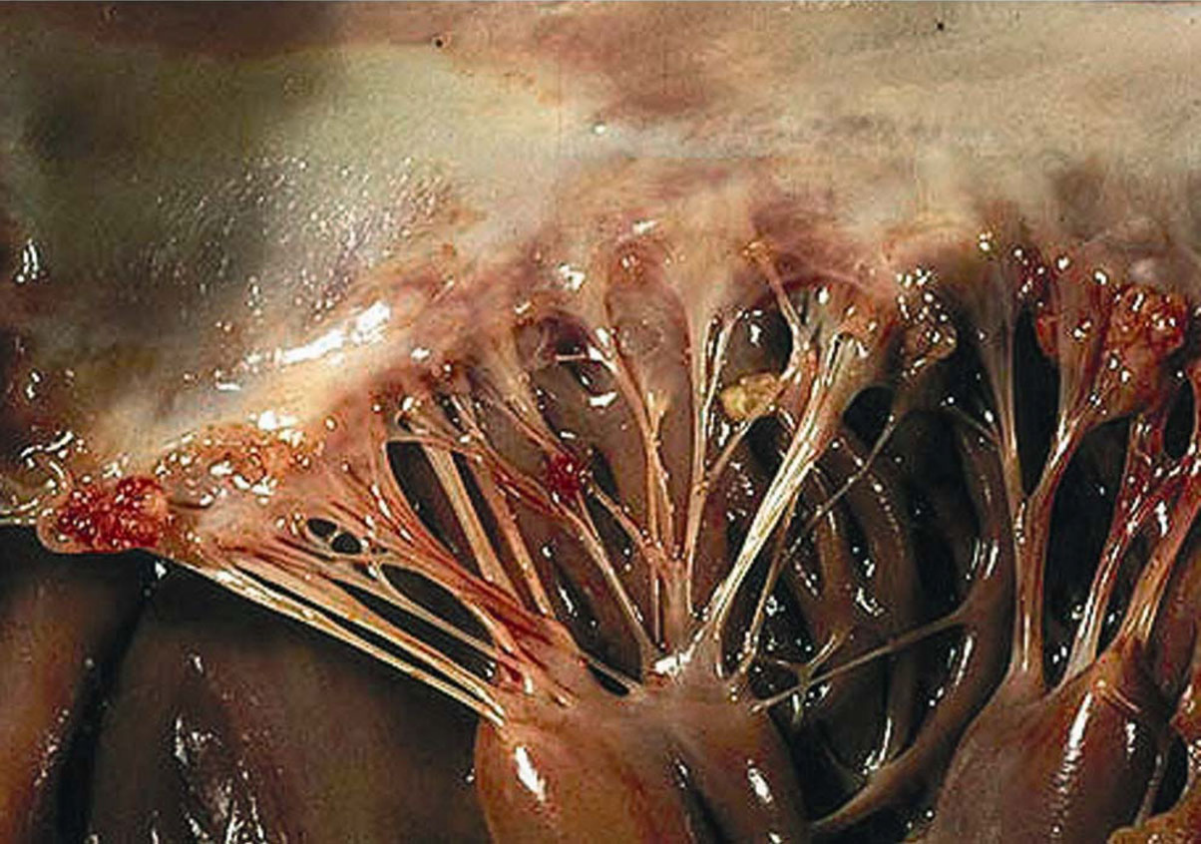
**Verrucous vegetations seen in Libman-Sacks endocarditis of the mitral valve**. The sterile fibrofibrinous vegetations seen in LS endocarditis of the mitral valve may vary in size and typically have a wart-like morphology. They can be found near the edge of the leaflets along the line of closure; both on the atrial and ventricular sides of the leaflets. They can even be found on the chordae and the endocardium. In this case several microthrombi are present on the free edge of the leaflet and on the chordae. *Reproduced with permission from Dr. S. Gonzalez. Copyright 2009, department of Pathology, Pontifical Catholic University of Chile, Santiago, Chile*.

In 1924 Libman and Sacks first described four cases of non-bacterial verrucous vegetative endocarditis [1]. The sterile verrucous lesions of Libman-Sacks (LS) endocarditis (Fig 1) show a clear predisposition for the mitral and aortic valves and are nowadays seen as both a cardiac manifestation of systemic lupus erythematosus (SLE) and, more recently, of the antiphospholipid syndrome (APS) [2-5].

SLE is an autoimmune disorder resulting in multi-organ inflammatory damage. Over the last decades with prolonged survival and improvement in diagnostic techniques, particularly in echocardiography, cardiac disease associated with SLE has become more apparent [6,7]. A recent echocardiographic study in patients with SLE revealed that LS vegetations can be found in approximately 11% of patients with SLE [8]. In 63% of these patients with vegetations the mitral valve was involved [8]. Earlier echocardiographic studies reported a higher prevalence of LS vegetations in patients with SLE, ranging from 53% to 74% [9,10].

Antiphospholipid syndrome (APS) has been defined as venous or arterial thrombosis, recurrent fetal loss, or thrombocytopenia accompanied by increased levels of antiphospholipid antibodies (aPLs) (i.e anticardiolipin antibodies and the lupus anticoagulant) [11-14]. This syndrome can be either primary or secondary to an underlying condition (most commonly SLE) [11-14]. An echocardiographic study in patients with primary APS showed that approximately one third of these patients have LS valvular lesions [4]. SLE is frequently accompanied by the presence of aPLs, which is associated with a higher prevalence of valvular abnormalities in SLE patients [5,15].

Although typically mild and asymptomatic, LS endocarditis can lead to serious complications, including superimposed bacterial endocarditis, thromboembolic events, such as stroke and transient ischaemic attacks, and severe valvular regurgitation and/or stenosis requiring surgery.

The literature on mitral valve surgery for mitral regurgitation (MR) caused by LS endocarditis is comparatively sparse. In this study we report two cases of mitral valve repair and two cases of mitral valve replacement for MR caused by LS endocarditis. In addition, we provide a systematic review of the English literature on mitral valve surgery for MR caused by LS endocarditis.

## Case Reports

We analyzed our institution's mitral valve surgery database and found four patients who underwent mitral valve surgery for MR caused by LS endocarditis in the period 1995-2008.

### Patient 1

A 49-year-old Caucasian man presented at our institution with SLE that had been diagnosed originally in August 1996. Manifestations of his disease included arthritis, a rash on sun-exposed skin, and skin lesions resembling urticaria. Laboratory findings are shown in Table 1. A skin biopsy revealed urticarial vasculitis. There was no evidence of cerebral or renal involevement. His therapy for SLE required long-term plaquenil and prednisone. In September 1997 the patient was admitted with progressive exertional dyspnoea, cardiac decompensation, and a blowing systolic murmur at the apex radiating to the left axilla. Transthoracic (TTE) and transesophageal echocardiography (TEE) revealed severe MR with thickened mitral valve leaflets and a small vegetation on the posterior mitral valve leaflet. Repeated blood cultures were negative and there was no other evidence of infective endocarditis. The patient was recompensated with diuretics and discharged. Echocardiographic follow-up over the following months revealed a rapid increase in left ventricular diameters and normal left ventricular (LV) function. Results of cardiac catherization are shown in Table 1. The patient underwent mitral valve repair in March 1998. Intraoperative inspection showed slightly thickened, but otherwise surprisingly normal leaflets. A small perforation was found in the P2 section of the posterior leaflet. Preoperatively a small vegetation was found near this location. Although rare and more often seen in infectious endocarditis, leaflet perforation in LS endocarditis has been reported before [16]. This patient's history did not reveal any documented thromboembolic events. A quadrangular resection of the P2 section of the posterior mitral valve leaflet was performed, followed by implantation of a 32 mm Carpentier-Edwards Classic annuloplasty ring. Microscopic examination of the excised mitral valve segment revealed myxoid degeneration and no evident signs of inflammation. Although evidence of LS endocarditis could not be found microscopically, the diagnosis was made based on the clinical features, laboratory findings, and echocardiographic appearance. The patient's recovery from surgery was uneventful, and he was discharged on the seventh postoperative day. Echocardiographic follow-up revealed stable slight MR from April 1998 through January 2009. When last seen in March 2009, the patient was doing well, except for a mild degree of dyspnoea.

**Table 1:** Preoperative baseline characteristics of four patients with MR caused by LS endocarditis

Patient	1	2	3	4
Gender	Male	Male	Female	Female
Age (years)	49	56	28	22
SLE/APS	SLE	SLE	APS	APS
Years of SLE/APS	1.5	4	1	0.5
Steroids	yes	yes	no	no
Valve Lesion	MR	MR	MR	MR
NYHA class	IV	I	III	I
Echocardiography				
-MR grade	4+	4+	4+	2+
-LV function	normal	normal	normal	normal
Cardiac Catheterization				
-Coronary artery disease	no	no	no	NA
-PAP (mmHg) (N: 15-30/3-12 mmHg)	34/6	41/18	32/21	NA
-PCWP (mmHg) (N: 1-10 mmHg)	10	18	21	NA
-LVEDP (mmHg) (N: 3-12 mmHg)	10	18	19	NA
-Cardiac Output (L/min/m^2^) (N: 2.6-4.2 L/min/m^2^)	2.74	3.20	4.30	NA
Laboratory tests				
-Repeated blood cultures	neg	neg	neg	neg
-CRP (mg/l) (N: 0-5 mg/l)	38	60	3	34
-White blood cell count (×10^9^/l) (N: 4.0-10.0 × 10^9^/l)	4.6	3.8	6.8	8.9
-Thrombocyte count (×10^9^/l) (N: 150-300 × 10^9^/l)	258	249	105	114
-Lupus anticoagulant (N: neg)	NA	NA	pos	pos
-Anti-cardiolipin Ab (IgG) (U/ml) (N: <10 U/ml)	<10	25	>100	53
-Anti-cardiolipin Ab (IgM) (U/ml) (N: <10 U/ml)	<10	<10	<10	<10
-Complement C3 (g/l) (N: 0.90-1.80 g/l)	0.39	0.77	1.19	1.57
-Complement C4 (g/l) (N: 0.10-0.40 g/l)	0.13	0.19	0.31	0.39
-Anti-Nuclear Antibody (ANA) titer (N: <20)	320	>640	40	40
-Anti-ds-DNA Ab (Farr-assay) (U/ml) (N: <10 U/ml)	11	154	<3	5
-Extractable Nuclear Antigens (ENA) (N: neg)	NA	neg	neg	neg

Ab, antibodies; APS, anti-phospholipid syndrome; CRP, C-reactive protein; ds-DNA, double stranded DNA; LS, Libman-Sacks; LV(EDP), left ventricular (end-diastolic pressure); MR, mitral regurgitation; N, normal values; NA, not available; NYHA, New-York heart association; PAP, pulmonary artery pressure; PCPW, pulmonary capillary wedge pressure; SLE, systemic lupus erythematosus

### Patient 2

**Figure 2:**
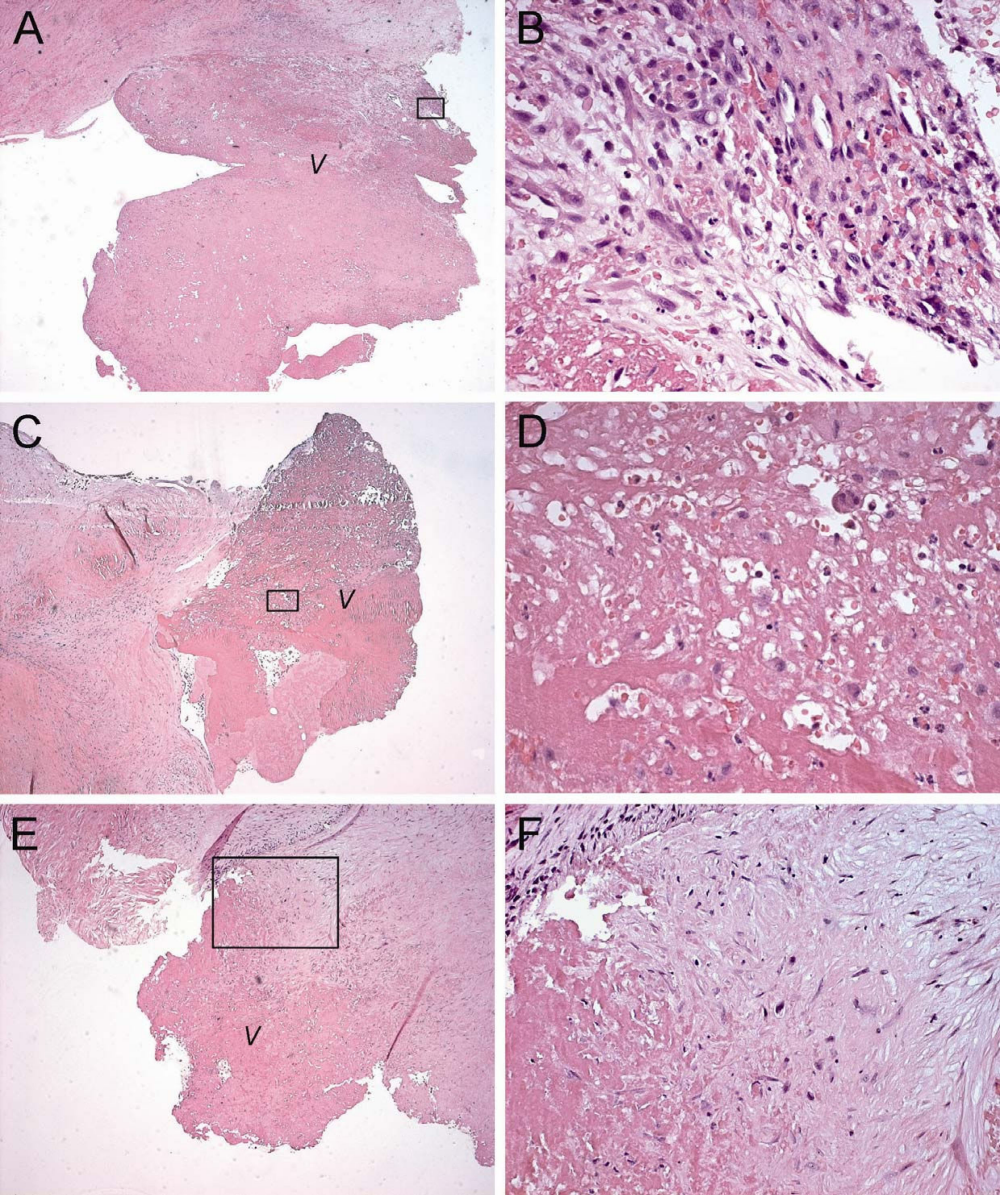
**Microscopic histopathological examination of excised mitral valve tissue in patient 2 (A,B), 3 (C,D), and 4 (E,F)**. **(A) **Photomicrograph of patient 2. Haematoxylin and Eosin (HE) stain of the atrial surface of the excised mitral valve anterior leaflet. Orginal magnification × 25. **(B) **Magnified section of A. Original magnification × 400. Fibrinoid changes and neovascularization at the base of the vegetation. The vegetation consists of fibrin-platelet thrombi and shows signs of acute and chronic inflammation with neutrophil and mononuclear cell infiltration. **(C) **Photomicrograph of patient 3. HE stain of the atrial surface of the excised mitral valve posterior leaflet. Orginal magnification × 50. **(D) **Magnified section of C. Original magnification × 400. Fibrinoid and hyaline changes at the base of the vegetation. The vegetation itself shows signs of fibroblastic organization of fibrin-platelet thrombus and an inflammatory infiltrate with neutrophils. **(E) **Photomicrograph of patient 4. HE stain of the atrial surface of the excised mitral valve posterior leaflet. Orginal magnification × 50. **(F) **Magnified section of E. Original magnification × 200. Fibrinoid and myxoid degenerative changes at the base of the vegetation. The vegetation shows signs of organization of fibrin-platelet thrombus without an evident inflammatory reaction. Sporadically, several neutrophils and mononuclear cells can be found in this section. *Black transparant rectangles outline magnified sections shown in the right-hand column. V: vegetation*.

A 56-year-old Caucasian man presented at our institution with severe SLE that had been diagnosed originally in July 2003. Manifestations of his disease included arthritis, pericarditis, and pleuritis without any evidence of skin, cerebral or renal involvement. Laboratory findings are shown in Table 1. His therapy for SLE required long-term prednisone, plaquenil and azathioprine. On routine examination in 2006 the patient appeared to have a blowing systolic murmur at the apex radiating to the left axilla. Transthoracic echocardiography (TTE) revealed mitral valve thickening with focal vegetations and severe MR. Repeated blood cultures were negative and there was no other evidence of infectious endocarditis. Results of cardiac catheterization are shown in Table 1. The patient underwent mitral valve replacement with a 31 mm St. Jude mechanical prosthesis in October 2007. The excised mitral valve was thickened and fibrotic with focal vegetations. Microscopic pathologic examination of the excised mitral valve revealed fibrosis, neovascularization, and vegetations with fibrin-platelet thrombi and evident inflammatory cell infiltration (Fig 2A,B). LS endocarditis of the mitral valve was confirmed. The patient's recovery from surgery was uneventful, and he was discharged on the seventh postoperative day. Echocardiographic follow-up revealed no MR. When last seen in April 2009, the patient was doing well.

### Patient 3

**Figure 3:**
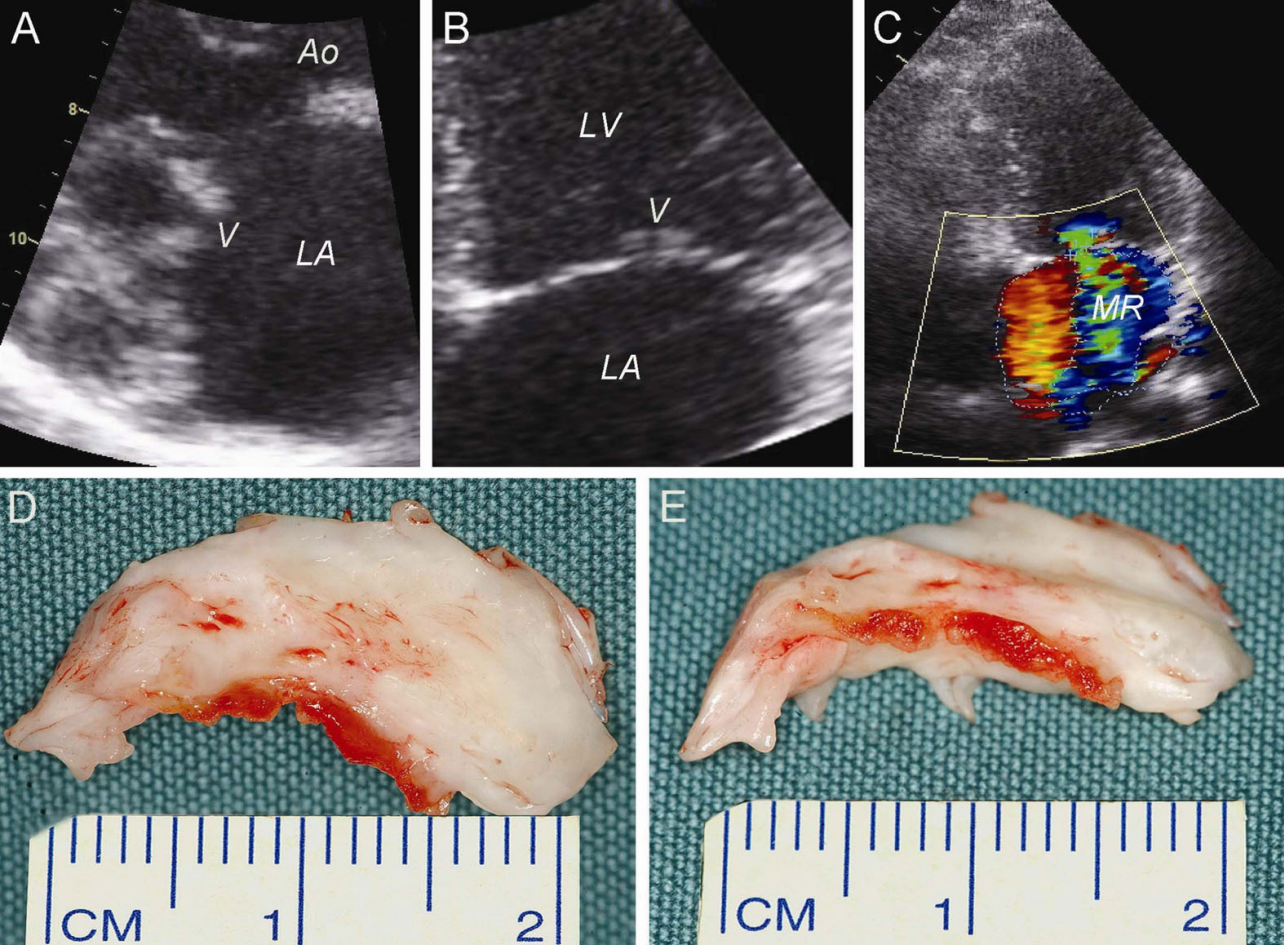
**Two-dimensional TTE examination and intra-operative inspection of the mitral valve in patient 3**. **(A) **Parasternal long-axis view, systolic. **(B) **Apical four-chamber view, systolic. Morphologic examination of the mitral valve leaflets in both views revealed several structural abnormalities, such as leaflet thickening and vegetations on the edges of both leaflets. **(C) **Severe MR as determined by jet area (13.4 mm^2^) divided by left atrial area (25.9 mm^2^) (= 52%) and the vena contracta width (= 6 mm, not shown). **(D) **Superior view of the excised posterior mitral valve leaflet (as seen from the left atrium). **(E) **Frontal view of the excised posterior mitral valve leaflet. Both views show marked thickening and calcification of the posterior mitral valve leaflet and several thrombotic vegetations on the edge of the leaflet. *Ao: aorta, LA: left atrium, LV: left ventricle, MR: mitral regurgitation and V: vegetations*.

A 28-year-old Caucasian woman was referred to our institution in October 2006 with arthralgias and intermittent haemoptysis. She had a missed abortion earlier that year, when she was nine weeks pregnant. Laboratory findings are shown in Table 1. The patient was diagnosed with primary APS. In November 2006 she presented with exertional dyspnoea and a blowing systolic murmur at the apex radiating to the left axilla. Transthoracic echocardiography (TTE) revealed mitral valve leaflet thickening with small vegetations on the edges of both leaflets (Fig 3A,B) and severe MR with backflow into the pulmonary veins (Fig 3C). Repeated blood cultures were negative and there was no other evidence of infectious endocarditis. Results of cardiac catheterization are shown in Table 1. The patient underwent mitral valve replacement in October 2007. Intraoperative inspection revealed thickened and fibrotic mitral valve leaflets with focal vegetations (Fig 3D,E). Therefore, mitral valve repair was not considered possible and the mitral valve was replaced with a 31 mm St. Jude mechanical prosthesis. Microscopic pathologic examination of the excised mitral valve revealed myxoid and hyaline degeneration, fibrosis, and vegetations with fibrin-platelet thrombi and evident inflammatory cell infiltration (Fig 2C,D). LS endocarditis of the mitral valve was confirmed. The patient's recovery from surgery was uneventful, and she was discharged on the seventh postoperative day. Echocardiographic follow-up revealed no MR. When last seen in June 2009, the patient was doing well.

### Patient 4

**Figure 4:**
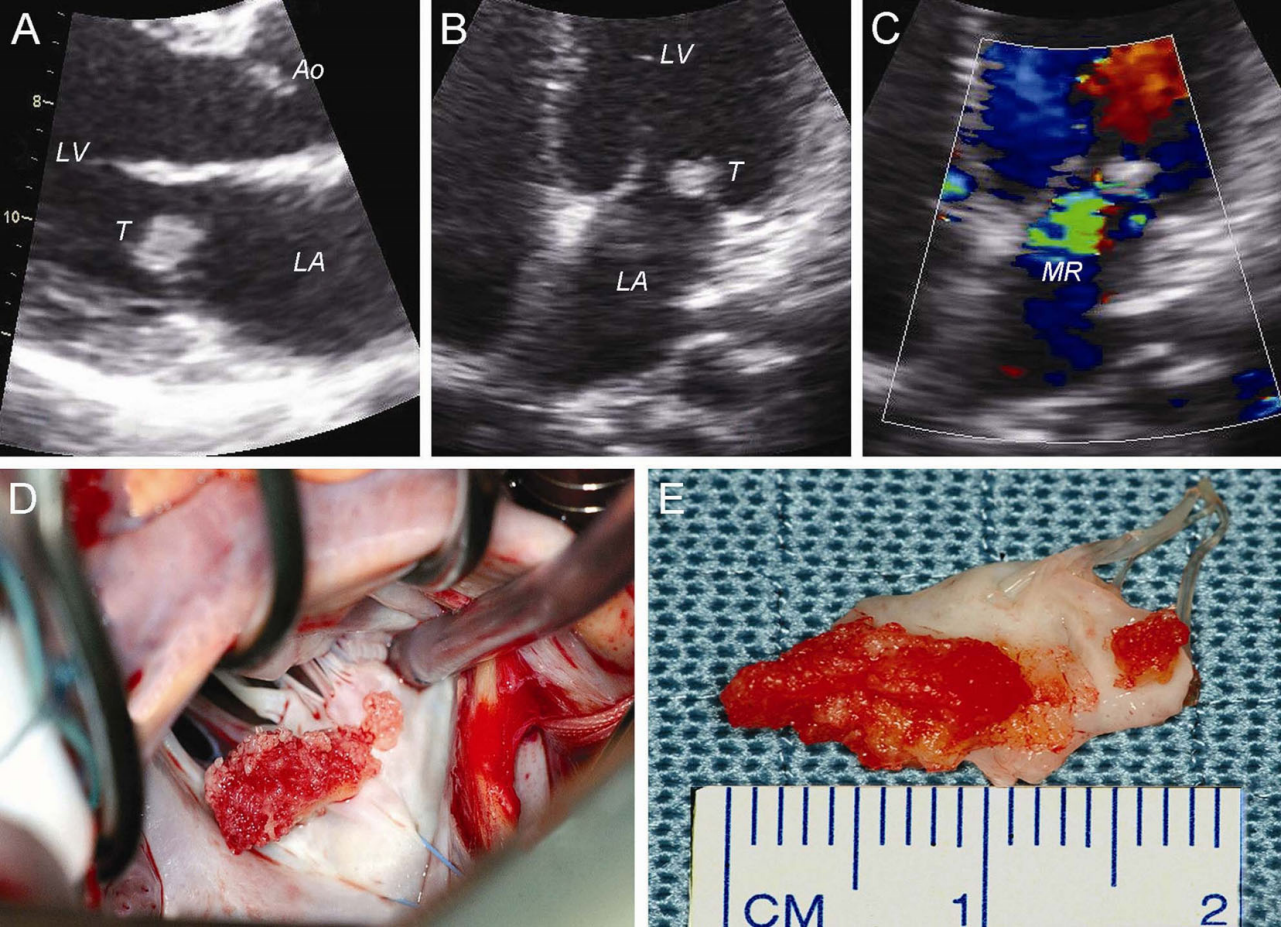
**Two-dimensional TTE examination and intra-operative inspection of the mitral valve in patient 4**. Morphologic examination of the mitral valve in both views revealed a 0.8 × 1.0 cm tumor on the posterior mitral valve leaflet; **(A) **Parasternal long-axis view, mid-diastolic; **(B) **Apical four-chamber view, end-diastolic. **(C) **Mild-to-moderate MR as determined with colour-Doppler TTE; apical four-chamber view, systolic. **(D) **Intra-operative inspection of the mitral valve (transseptal approach): a verrucous thrombotic tumor was found on the P2 section of the posterior mitral valve leaflet. **(E) **The verrucous thrombotic tumor was removed with a quadrangular resection of P2. *Ao: aorta, LA: left atrium, LV: left ventricle, MR: mitral regurgitation and T: tumor*.

A 22-year-old Hispanic woman with a history of hypothyreoidism was referred to our institution in October 2007 after a transient ischemic attack of the right cerebral hemisphere with temporary left hemiplegia. Routine trans-thoracic echocardiography revealed a tumor with a diameter of approximately 1 cm on the atrial side of the posterior mitral valve leaflet (Fig 4A,B) as the source of this thrombo-embolic event. In addition, a normal LV function and moderate (grade 2+) MR was found (Fig 4C). Repeated blood cultures were negative and there was no other evidence of infectious endocarditis. Based on her history and the echocardiographic appearance of the tumor the initial working diagnosis was papillary fibroelastoma. Laboratory findings are shown in Table 1. The patient was diagnosed with primary APS. Subsequently, LS endocarditis of the mitral valve was considered as an alternative diagnosis. To prevent future thrombo-embolic events the patient was accepted for mitral valve surgery. Cardiac catherization was not performed. The patient underwent mitral valve repair in March 2008. Intraoperative inspection showed a large verrucous tumor on the atrial side of the P2 section of the posterior mitral valve leaflet (Fig 4D). A quadrangular resection of the P2 section of the posterior mitral valve leaflet was performed (Fig 4E), followed by implantation of a 28 mm Cosgrove-Edwards annuloplasty ring. Microscopic examination of the excised mitral valve segment revealed myxoid degeneration and large vegetations with fibrin-platelet thrombi, but without an evident inflammatory infiltrate (Fig 2E,F). The initial working diagnosis of papillary fibroelastoma could not be confirmed on microscopic examination. A definite diagnosis of LS endocarditis was made. The patient's recovery from surgery was uneventful, and she was discharged on the seventh postoperative day. Echocardiographic follow-up after 1.5 years revealed no recurrence of MR. When last seen in September 2009, the patient was doing well.

## Systematic Review

We systematically reviewed the literature on mitral valve surgery for (isolated) MR caused by (SLE and/or APS related) LS endocarditis (Table 2). We performed separate Medline (PubMed), EMBASE, and Cochrane database queries with the following text and keywords: "libman-sacks endocarditis, mitral", "antiphospholipid syndrome, mitral", and "non-bacterial thrombotic endocarditis, mitral". All papers were considered irrespective of their quality or the journal in which they were published. We then used strict criteria. Titles and abstracts were screened and relevant papers were selected. All papers with a case report or a series of case reports on mitral valve surgery for (isolated) MR caused by LS endocarditis were included. Reports not written in English were excluded, as well as reports without a clear description of MR etiology and/or mitral valve pathology. In addition, cases of mitral valve surgery for mitral stenosis (MS) (4 cases) or combined MR and MS (11 cases) caused by LS endocarditis were excluded. Although these exclusions may be seen as a limitation, we believe it is a particular strength of this study, since it generated a "clean" cohort of patients that underwent mitral valve surgery for (isolated) MR caused by Libman-Sacks endocarditis.

**Table 2:** Systematic review of the English literature on mitral valve surgery for (isolated) MR caused by Libman-Sacks endocarditis^a^

Reference	Year published	Gender/ Age (years)	SLE and/ or APS	Years of SLE and/or APS	Steroids	MR grade^b^	MVR/MVP	Surgical procedure	Follow-up
Myerowitz *et al *[48]	1974	F/22	SLE	3	yes	4+	MVR	Reis-Hancock porcine bioprosthesis	alive 2 months post-op
Murray *et al *[49]	1975	F/43	SLE	2	yes	4+	MVR	Beall Surgitoul mechanical prosthesis	alive 6 months post-op
Paget *et al *[50]	1975	F/18	SLE	4	yes	4+	MVR	Porcine xenograft (bioprosthesis)	alive 4 months post-op
Kinney *et al *[51]	1980	F/27	SLE	0.3	no	4+	MVR	Hancock porcine bioprosthesis	NR
Rawsthorne *et al *[52]	1981	M/51	SLE	21	no	4+	MVR^c^	Hancock porcine bioprosthesis	NR
Brennan *et al* [53]	1983	F/20	SLE	2	yes	4+	MVR	Björk-Shiley mechanical prosthesis	CVA 17 months post-op
Rozman *et al* [54]	1986	M/43	SLE	2	yes	NR	MVR	Starr-Edwards mechanical prosthesis	alive 4 years post-op
Moynihan *et al* [55]	1988	F/54	SLE	2	yes	4+	MVR^c^	Carpentier-Edwards porcine bioprosthesis	alive 26 months post-op
Straaton *et al* [56]	1988	F/22	SLE	<1	no	4+	MVR^d^	Carpentier-Edwards heterograft (bioprosthesis)	NR
		F/67	SLE	10	yes	2+/3+	MVR^c^	St. Jude mechanical prosthesis	died intraoperatively
Ferraris *et al* [57]	1990	M/34	SLE	9	yes	4+	MVR	Carpentier-Edwards bioprosthesis	alive 2.5 years post-op
Alvarez *et al* [58]	1994	F/42	APS	1	no	2+/3+	MVR	Medtronic Hall mechanical prosthesis	NR
Kalangos *et al* [40]	1995	F/28	SLE	0.5	yes	4+	MVP	Resection of the prolapsed posterior commissure and restoration with an autologous pericardial patch; sliding plasty of the anterior leaflet; transposition of secondary chordae to the commisure; Carpentier- Edwards annuloplasty ring	no MR recurrence 1 year post-op
Chauvaud *et al *[41]	1995	F/17	SLE	5	yes	4+	MVP	Posterior leaflet enlargement with pericardial patch and Carpentier-Edwards annuloplasty ring	MS 6 months post-op due to calcification
							redo MVR	Cryopreserved homograft	alive 1 year post-op
Shahian *et al* [59]	1995	F/29	APS	0	yes	4+	MVR	St. Jude mechanical prosthesis	alive 2 years post-op
Gordon *et al* [46]	1996	M/37	SLE, APS	>2	yes	1+/2+	MVR^c^	Carpentier-Edwards porcine bioprosthesis	recurrent A-V fistula
							redo MVR	Carpentier-Edwards porcine bioprosthesis	died 1 month post-op
Morin *et al *[33]	1996	F/40	SLE	12	yes	4+	MVR	St. Jude mechanical prosthesis	alive 2 weeks post-op
East *et al *[60]	2000	F/51	SLE, APS	NR	no	4+	MVR^e^	not further specified	NR
		F/49	SLE, APS	7	yes	4+	MVR	not further specified	NR
Hakim *et al *[42]	2001	F/23	SLE	8	yes	4+	MVR	St. Jude mechanical prosthesis	alive >1 year post-op
		F/54	SLE	7	yes	4+	MVP^f^	not further specified	MR 29 months post-op
							redo MVR	St. Jude mechanical prosthesis	alive >5 years post-op
		F/64	SLE	2	yes	4+	MVP^e^	Quadrangular resection of the posterior leaflet and Duran annuloplasty ring	alive >3 years post-op
Kato *et al *[43]	2001	F/52	APS	NR	no	4+	MVP	Kay's annuloplasty and Cosgrove annuloplasty ring	alive 3 months post-op
Mottram *et al* [23]	2002	M/50	APS	0	no	0/1+	MVP	Removal of two mitral valve masses	alive 9 weeks post-op
da Silva *et al* [47]	2003	F/54	SLE, APS	13	yes	4+	MVR	Bioprosthesis (not further specified)	bioprosth. thrombosis and death 9 months post-op
Schneider *et al *[44]	2003	M/23	SLE	NR	NR	4+	MVP	not further specified	NR
Georghiou *et al *[61]	2003	F/44	SLE	1	yes	3+	MVR^c,d^	St. Jude mechanical prosthesis	alive 6 months post-op
Bordin *et al *[62]	2003	F/57	SLE, APS	3	NR	4+	MVR	Mechanical prosthesis (not further specified)	MI 2 days post-op
Berkun *et al* [63]	2004	F/48	APS	NR	yes	3+/4+	MVR	Carbomedics mechanical prosthesis	MR 90 months post-op
							redo MVR	not further specified	died 6 months post-op
		F/73	APS	NR	yes	3+/4+	MVR	Hancock porcine bioprosthesis	died 13 months post-op
		F/47	APS	NR	yes	3+/4+	MVR^c^	Carbomedics mechanical prosthesis	splenic embolus 3 and CVA 10 months post-op alive 42 months post-op
		F/38	SLE, APS	NR	yes	3+/4+	MVR	Carbomedics mechanical prosthesis	alive 32 months post-op
		F/51	SLE, APS	NR	yes	3+/4+	MVR	Carbomedics mechanical prosthesis	alive 33 months post-op
Fernández *et al *[45]	2005	F/36	SLE	8	NR	4+	MVP	not further specified	mild MR 1 year post-op
Taguchi *et al* [24]	2006	F/34	SLE	9	yes	0/1+	MVP	Resection of A3 and attached chordae (en-bloc); restoration of the anterior leaflet; 4 PTFE neochordae	no MR recurrence 6 months post-op
Einav *et al *[64]	2007	F/28	SLE, APS	4.5	yes	4+	MVR^c,d^	Mechanical prosthesis (not further specified)	NR
Takayama *et al *[16]	2008	M/58	SLE, APS	0	no	3+/4+	MVP	Valvuloplasty and annuloplasty (not further specified)	NR
Bouma *et al*	2010	M/49	SLE	1.5	yes	4+	MVP	Quadrangular resection of the posterior stable trace MR leaflet (P2) and Carpentier-Edwards annuloplasty ring	stable trace MR 11 years post-op
		M/56	SLE	4	yes	4+	MVR	St. Jude mechanical prosthesis	alive >1.5 years post-op
		F/28	APS	1	no	4+	MVR	St. Jude mechanical prosthesis	alive >1.5 years post-op
		F/22	APS	0.5	no	2+	MVP	Quadrangular resection of the posterior leaflet (P2) and Cosgrove-Edwards annuloplasty ring	no MR recurrence 1.5 years post-op

A-V, atrioventricular; APS, antiphospholipid syndrome; CVA, cerebrovascular accident; F, female; M, male; MI, myocardial infarction; MR, mitral regurgitation; MS, mitral stenosis;MVP, mitral valve plasty; MVR, mitral valve repair; NR, not reported; PTFE, polytetrafluorethylene; SLE, systemic lupus erythematosus

^a^reports not written in English or reports of mitral valve surgery in patients with SLE and/or APS without a description of MR etiology and mitral valve pathology were excluded; cases of MS (n = 4) or combined MR/MS (n = 11) caused by Libman-Sacks endocarditis were also excluded

^b^MR severity grading: 0, no or trace MR; 1+, mild MR; 2+, moderate MR; 3+, moderate-to-severe MR; 4+, severe MR

^c^including aortic valve replacement (AVR)

^d^including tricuspid valve plasty (TVP)

^e^including CABG

^f^including aortic valve plasty (AVP)

## Discussion

Nowadays LS endocarditis is seen as a cardiac manifestation of both SLE and APS [2-5]. LS endocarditis is usually typically mild and asymptomatic, but can lead to serious complications, such as superimposed bacterial endocarditis, thromboembolic events, and valvular regurgitation and/or stenosis requiring surgery. The mitral valve is most commonly affected [8,10]. The presence of APLs in patients with SLE is related to a higher prevalence of valvular abnormalities [5,15], which suggests a possible role for APLs in the pathogenesis.

### Pathogenesis

At this point the exact pathogenesis of LS endocarditis is still unclear. The initial insult to the valve, which causes endothelial damage and elicits the pathogenetic sequence of events, has not yet been identified. However, LS endocarditis has been assumed to involve the formation of fibrin-platelet thrombi on the altered valve, the organization of which leads to valve fibrosis, edema, diffuse thickening, mild inflammatory changes, valve distortion, scarring, and subsequent valvular dysfunction [5,7,17-19]. Both valve thickening and formation of vegetations represent different stages of the same pathological proces [5]. Immunologic injury has been postulated as a possible initiating insult, since immunofluorescent microscopy revealed deposition of immunoglobulins and complement on affected valves [5,19]. Rather than playing a more direct pathogenetic role, aPLs are thought to promote thrombus formation on the endothelium of valves already compromised by immune complex deposition, leading to further valvular damage and inflammation [5,8,15,17,18,20,21].

### Microscopy

Valvular LS lesions are microscopically characterized by fibrin deposits at various stages of fibroblastic organization, neovascularization, occasional haematoxylin bodies, and by a variable extent of inflammation with mononuclear cell infiltration [6,7]. Valvular lesions change over time [10] and the end-stage or healed form of LS verrucous endocarditis is a fibrous plaque, sometimes with focal calcification [5]. If the lesions are extensive enough, their healing may be accompanied by marked scarring, thickening, and deformity of the valve [5].

### LS Endocarditis versus Non-Bacterial Thrombotic Endocarditis (NBTE)

Microscopically, the mitral valve vegetations seen in SLE are distinct from those seen in (primary) APS. A rather remarkable difference is the absence (or minimal extent) of inflammatory cell infiltration in (primary) APS [5,22]. To emphasize this difference some authors prefer to use the term NBTE for the valve lesions seen in primary APS instead of the term LS endocarditis. However, others (including the authors of this study) prefer to use the term LS endocarditis, because the two underlying diseases are both auto-immune phenomena, are often interrelated (APS secondary to SLE), and probably share a (partially) similar pathologic pathway in causing valve lesions.

### Differentiation from infective endocarditis and intracardiac tumors

Due to its asymptomatic nature establishing a diagnosis of LS endocarditis can be rather difficult. This is further complicated by the fact that the condition can mimick intracardiac tumors [23-25] and bacterial endocarditis ("pseudoinfective" endocarditis) [26] or may coexist with (superimposed) bacterial endocarditis (also known as "double-decker" endocarditis) [7,26,27].

The modified Duke criteria can be useful in helping differentiate between true infective endocarditis and LS endocarditis [28]. Helpful laboratory markers in distinguishing infective endocarditis from LS endocarditis are the white blood cell count (elevated in infective endocarditis and often decreased in LS endocarditis), C-reactive protein levels (elevated in infective endocarditis and relatively low in LS endocarditis), aPL levels (normal in infective endocarditis and moderate to high in LS endocarditis), and (repeated) blood cultures (positive in infective endocarditis and negative in LS endocarditis) [21,29]. Echocardiographically, LS vegetations appear as valve masses of varying size and shape with irregular borders and echodensity, they are firmly attached to the valve surface and exhibit no independent motion [8]. Contrary to the vegetations of infective endocarditis, which typically exhibit independent motion [30].

As previously demonstrated [23-25] and as we showed in patient 4, differentiation from intracardiac tumors can also be difficult. Although LS vegetations are usually typically sessile, wartlike, and small, varying from pinhead size to 3-4 mm [5], they can become rather large making them difficult to distinguish (echocardiographically) from a typical mitral valve tumor such as papillary fibroelastoma. On echocardiography papillary fibroelastoma usually arise via a pedicle from mitral valve tissue or adjacent endocardium, and have a characteristic frond-like appearance [31]. A remarkable feature that LS vegetations do not posses.

Recently, a prospective randomized controlled study showed that TEE was superior to TTE in diagnosing LS endocarditis [32]. Nevertheless, establishing the diagnosis remains challenging.

### Treatment with corticosteroids and anticoagulation

Corticosteroids do not prevent LS endocarditis, but they facilitate healing of LS lesions over time by decreasing the amount of inflammation [5,33-35]. However, they can increase fibrosis and scarring, ultimately worsening valvular damage and dysfunction [5,33-35]. Nonetheless, appropriate steroid therapy to control SLE disease activity is important.

The risk of thrombo-embolic events (mainly stroke and transient ischaemic attacks) is increased in LS endocarditis [8]. Current therapeutic guidelines for APS include thrombo-embolic prevention with long-term anticoagulation [5]. In addition, patients with LS endocarditis who have suffered a thrombo-embolic event are recommended to be on lifelong anticoagulation for prevention of future thrombo-embolic events [5]. Moreover, implantation of a mechanical valve requires anticoagulation and atrial fibrillation is frequently a concomitant condition necessitating anticoagulation in patients with severe MR. In other words, lifelong anticoagulation can often not be avoided in these patients.

### Mitral valve surgery

In most patients hemodynamically important valvular dysfunction can be controlled with conservative treatment (i.e immunosuppression, anticoagulation, endocarditis profylaxis, and specific heart failure treatment including ACE-inhibitors, beta-blockers, diuretics) [5,36,37]. However, if severe symptomatic valvular dysfunction persists mitral valve surgery may be required.

### Valve repair versus replacement and valve selection

In contemporary cardiac surgery mitral valve repair has become the mainstay of surgical treatment for most causes of MR. Particularly, in the last two decades there has been a gradual shift from mitral valve replacement to mitral valve repair for MR caused by a broad range of etiologies. Several general advantages of mitral valve repair over replacement include a lower operative mortality rate, higher survival rates, better maintainance of left ventricular function, a lower risk of endocarditis, a lower risk of thrombo-embolic complications, less use of lifelong anticoagulation, and lower costs [38,39].

In recent years mitral valve repair for significant MR due to LS endocarditis has been described in 10 patients (Table 2) [16,23,24,40-45]. Unfortunately the exact surgical repair techniques were not described in 4 of these patients [16,42,44,45]. In this report we added two mitral valve repair cases to the literature with a precise description of mitral valve pathology and mitral valve repair techniques. In both patients (one with SLE and one with primary APS) intraoperative macroscopic examination revealed only localized abnormalities with otherwise relatively normal leaflets. Therefore mitral valve repair was considered a good surgical option in these two patients. Echocardiographic and clinical follow-up of both patients after 11 and 1.5 years, respectively, showed excellent results and no recurrence of MR. To our knowledge, this is the longest follow-up ever described after mitral valve repair for MR caused by LS endocarditis.

Some studies suggest that results of mitral valve replacement (MVR) are usually superior to repair for LS endocarditis [41,42]. According to these studies, severe (ongoing) calcification and fibrosis lead to rapid recurrence of MR after repair with a subsequent reoperation and MVR [41,42]. In our systematic review we found two cases of MVR after initial repair for MR due to LS endocarditis [41,42]. In the first case severe mitral stenosis developed 6 months after mitral valve repair due to ongoing calcification [41] and in the second case severe MR recurred 29 months after repair [42].

Taken together, we believe mitral valve repair for LS endocarditis of the mitral valve can be justified in specific patients. If SLE and/or APS has been relatively stable (with immunosuppresive therapy) in a young patient, if intraoperative macroscopic examination reveals relatively normal leaflets with only localized abnormalities, and if repair seems feasible, then mitral valve repair is in our opinion certainly justified and probably the preferred surgical option. Especially in young females, who are likely to become pregnant in the near future, long-term anticoagulation is preferably avoided by mitral valve repair. However, as previously mentioned, anticoagulation is often still necessary in LS endocarditis and APS to prevent future thrombo-embolic events. In that case mitral valve replacement with preservation of the subvalvular apparatus may be a good surgical alternative.

In case of mitral valve replacement, prosthetic valve selection is highly individualized based on age and other factors. In patients with bleeding abnormalities the superiority of bioprosthetic valves over mechanical valves is clear. However, mechanical valve replacement is recommended in patients at low risk with anticoagulation and at high risk for bioprosthetic valve calcification. Although succesful placement of porcine bioprostheses in patients with LS endocarditis has been reported (Table 2), complications can arise. In our systematic review we found one case in which a bioprosthetic porcine valve had to be replaced, because it was affected by (rapid) calcification and valvulitis and subsequent perforation [46] and another case in which massive bioprosthetic thrombosis occurred [47]. Again stressing the importance of anticoagulation in this disease. In addition, (SLE associated) renal failure can accelerate bioprosthetic degeneration as a consequence of abnormal calcium and phosphate metabolism [40]. Therefore, a mechanical prosthesis may provide better results than a bioprosthesis for MR caused by LS endocarditis, even though mechanical prostheses carry a higher risk of thrombo-embolic complications.

Evidently, no definite consensus has been reached at this point as to whether or not these valves should be replaced or repaired and whether a mechanical prosthesis is more advantageous than a bioprosthesis.

## Conclusion

LS endocarditis should be strongly suspected when significant valve dysfunction, such as MR, develops during the course of SLE and/or APS. Differentiation from infective endocarditis and intracardiac tumors can be difficult, but is important and has different therapeutic implications. After establishing the diagnosis, periodic echocardiographic follow-up is recommended to detect detoriation of valvular function. When severe symptomatic MR requires surgery, mitral valve repair should always be considered. This report showed that mitral valve repair is feasible and effective in young patients with relatively stable SLE and/or APS and only localized mitral valve abnormalities caused by LS endocarditis. Both clinical and echocardiographic follow-up showed excellent mid- and long-term results.

## Abbreviations

aPLs: antiphospholipid antibodies; APS: antiphospholipid syndrome; LS: Libman-Sacks; LV: left ventricle; MR: mitral regurgitation; MS: mitral stenosis; MVP: mitral valve plasty; MVR: mitral valve replacement; NBTE: non-bacterial thrombotic endocarditis; SLE: systemic lupus erythematosus; TEE: transesophageal echocardiography; TTE: transthoracic echocardiography.

## Competing interests

The authors declare that they have no competing interests.

## Authors' contributions

WB collected the data, systematically reviewed the literature, and wrote the manuscript. TK was lead surgeon. TK, IH, IJH, ME, MB, AS, FZ, and MM participated in the design of the manuscript and they revised and critically reviewed the manuscript. All authors read and approved the final manuscript.
